# Soil from an Abandoned Manganese Mining Area (Hunan, China): Significance of Health Risk from Potentially Toxic Element Pollution and Its Spatial Context

**DOI:** 10.3390/ijerph17186554

**Published:** 2020-09-09

**Authors:** Xin Luo, Bozhi Ren, Andrew S. Hursthouse, Jonathan R. M. Thacker, Zhenghua Wang

**Affiliations:** 1 Hunan Provincial Key Laboratory of Shale Gas Resource Exploitation, Xiangtan 411201, China; luoxin@mail.hnust.edu.cn (X.L.); andrew.hursthouse@uws.ac.uk (A.S.H.); wzh@hnust.edu.cn (Z.W.); 2 School of Civil Engineering, Hunan University of Science and Technology, Xiangtan 411201, China; 3 School of Computing Engineering & Physical Sciences, University of the West of Scotland, Paisley PA1 2BE, UK; 4 School of Health & Life Sciences, University of the West of Scotland, Paisley PA1 2BE, UK; richard.thacker@uws.ac.uk

**Keywords:** abandoned manganese mine, potentially toxic element, spatial distribution, ecological risk assessment, source analysis, health risk assessment

## Abstract

This study assessed the significance and potential impact of potentially toxic element (PTE) (i.e., Mn, Pb, Cu, Zn, Cr, Cd, and Ni) pollution in the surface soil from an abandoned manganese mining area in Xiangtan City, Hunan Province, China, on the health of residents. The risks were sequentially evaluated using a series of protocols including: the geo-accumulation index (I_geo_), pollution load index (PLI), potential ecological risk index (RI), and implications for human health from external exposures using the hazard quotient (HQ), hazard index (HI) and carcinogenic risk (CR). The results revealed that Mn and Cd were the major pollutants in the soil samples. The ecological risk assessment identified moderate risks, which were mainly derived from Cd (82.91%). The results of the health risk assessment revealed that generally across the area, the non-carcinogenic risk was insignificant, and the carcinogenic risk was at an acceptable level. However, due to local spatial fluctuation, some of the sites presented a non-carcinogenic risk to children. The soil ingestion pathway is the main route of exposure through both non-carcinogenic and carcinogenic risks, with Mn being the major contributor to non-carcinogenic risk, with Cr and Cd the major contributors to carcinogenic risk. In addition, three pollution sources were identified through the Pearson correlation coefficient and principal component analysis (PCA), which included: a. mining activities and emissions from related transportation; b. natural background; c. agricultural management practices and municipal sewage discharge. The study provides information on the effects of spatial variation for the development of the abandoned mining areas and a useful approach to the prioritization of locations for the development and utilization of soil in these areas in China.

## 1. Introduction

The impact of pollution from a range of potentially toxic elements (PTEs) on humans and wider ecosystems remains a major concern for society [1]. Many PTEs are found in the soil and rocks in nature, but most significant are the releases into soil from various human activities [1], such as mining activities (mining and smelting), agricultural production (application of pesticides, herbicides), industrial activities, transport emissions, etc. [1,2,3]. Enriched levels can have a number of impacts such as creating wasteland, and affecting the growth and reproduction of biological organisms [1]. In addition, PTEs in the soil can enter the human body through the enrichment in the food chain, direct ingestion, dermal contact and inhalation, and eventually cause harm to the human body and cause various diseases [1,4,5]. Some relevant examples are the accumulation of Mn in the human body which can cause mental illness and even Parkinson’s syndrome [6]; excessive Cd can cause renal insufficiency and prostate disease [2] and the accumulation of Pb can lead to high circulation in the blood and damage the endocrine and immune systems [1,7]. Excess Cu can affect liver and kidney function and even be fatal [1,6]. Thus, where enrichment exists, it is important to evaluate the significance of the pollution and undertake a more detailed risk assessment [8,9].

The manganese mining area in Xiangtan City has been producing manganese ore since 1913, providing a significant manganese resource for the country [10], but recently, production has stopped. To re-use previously impacted land it is necessary to evaluate the pollution levels and risk to the environment in order to achieve faster and better development. There are already many studies on the risk assessment of soil from mining, urban use, and for food production [1,8,9]. However, studies on the impact of risk assessment on future land use and its spatial variation are not common. In addition, the use of the geo-accumulation index (I_geo_) can not only reflect the natural changes in the distribution of single PTEs, but also demonstrate the impact of human activities on pollution by PTEs. The pollution load index (PLI) directly apportions the contribution of a single PTE to the combined pollution across the study area. The potential ecological risk index (RI) is one of the most commonly used methods to evaluate ecological risk. This method takes into account the combined effects of the concentration of potentially toxic elements and toxic response factors.

Since there is no clear health risk assessment method in China, the method recommended by EPA for health risk assessment was adopted, which includes the hazard quotient (HQ), and hazard index (HI), and carcinogenic risk (CR). The use of multivariate analysis such as principal component analysis (PCA) allows relationships between variables to be highlighted and when combined with the Pearson correlation coefficient, presents a good method of source apportionment. Therefore, this study reports on the assessment of risk from the potential reuse of soil in an abandoned mining area, through the use of a series of environmental and human health risk assessment methods including geo-accumulation index (I_geo_), pollution load index (PLI), potential ecological risk index (RI), HQ, HI, CR. The Kriging interpolation method was used to derive the spatial distribution of PTEs in the soil in the study area using ArcGIS 10.3. This study extends our foundation work in the region supporting the re-development and utilization of soil in the abandoned manganese mining area in Xiangtan. The spatial assessment provides direction for soil development and utilization and serves as a template for risk assessment in other mining areas in China [5,10,11].

The main objectives of this study were to: (1) assess the potential ecological risks of the local environment and the health risks to local residents from external exposure in a sub region of the mining area; (2) evaluate the significance of the PTE levels in the soil in the study area; (3) demonstrate spatial variability and its significance; (4) identify the source of seven PTEs in the soil using statistical analysis.

## 2. Materials and Methods

### 2.1. Study Area

**Figure 1:**
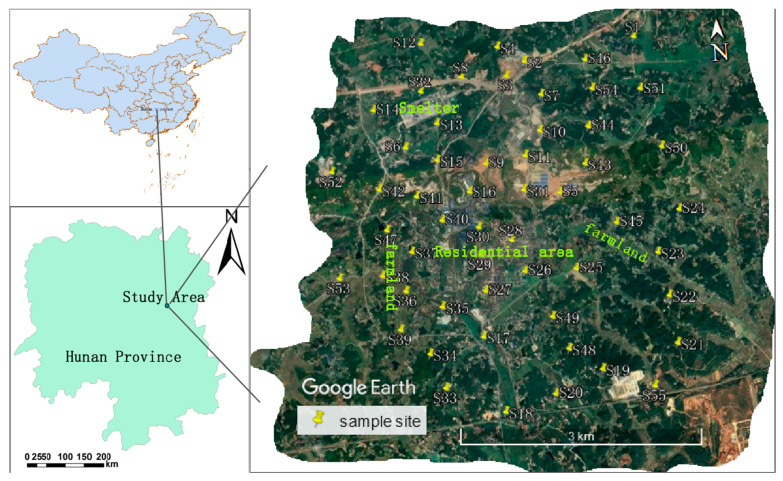
Distribution of sampling points in the study area.

The study area was located in the abandoned manganese mining area near Heling Town, Xiangtan City, Hunan Province, China (111°58′~113°05′ E, 27°21′~28°05′ N) (Figure 1). The study area is about 38.4 km^2^. The Xiangtan City region is in a humid subtropical monsoon climate zone. It is dry in the summer and autumn, and vulnerable to cold waves and high winds in winter and spring. The average temperature is 16.7–17.4 °C. Precipitation is high, but the seasonal distribution is uneven and the inter-annual variation is large. The annual precipitation is 1200–1500 mm [5,10,11]. The overall topographical characteristics of Xiangtan City presents a sharply undulating landscape with a maximum height of 150 m ASL with highest areas to the north, west, and south, and low lying areas in the central region and to the east. The Xiangtan City region is rich in mineral resources with the Mn-mineral deposits that are dominated by sedimentary manganese carbonate and secondary manganese oxide ore, as well as some non-metallic minerals, such as limestone, dolomite, coal, and gypsum [12]. We previously demonstrated the significance of surface processes in other locations of the mining region from the dispersal by wind blow and surface run off of residues close to former mining extraction points [5] and extended the work to mixed land use areas close to smelting and mining areas where farming had developed post-closure, to assess the impact of residual materials on the significance of exposure through the food chain [11]. This study identifies a residential location in the region, with external exposure from a range of sources, including mineral processing operations to provide a case to identify potential health impact from surface materials through external exposure (inhalation, dermal contact) pathways.

### 2.2. Sample Collection

A total of 55 surface (0–20 cm) soil samples were collected in the study area between September and October 2018 (Figure 1). The samples were collected from the representative land uses to include agricultural soil, roadside soil, woodland soil, and disturbed soil near industrial operations. A total of five sub-samples were collected at each location based on a 5 m radius circle (points at east, west, south, north, and center) and mixed into a 1 kg composite sample [13]. The bulk samples were sealed in polyethylene bags and immediately sent to the laboratory. After removing the obvious debris such as wood chips, crushed stones, and animal and plant residues, the soil samples were air-dried at room temperature (20~25 °C), and then ground through a 200-mesh stainless steel sieve. The processed samples were packed in polyethylene bags, labeled, and stored in the refrigerator until they were analyzed [14].

### 2.3. Sample Analysis

The samples were digested with HNO_3_+HF+HClO_4_. Weighed 0.5 g of soil sample was placed in a Teflon beaker, and 15 mL HNO_3_+HF+HClO_4_ was added and digested at 85 °C until a clear solution was obtained [15]. It was then filtered through Whatman No.42 filter paper (<2.5 μm) and then diluted to 50 mL with deionized water. The concentration of Mn, Pb, Cu, Zn, Cr, Cd, and Ni were determined by inductively coupled plasma mass spectrometry (ICP–MS, Finnigan MAT Element 2, ThermoFisher, Bremen DE). In sample detection, the limits were Mn (0.054 μg/kg), Pb (0.01 μg/kg), Cu (0.1 μg/kg), Zn (0.5 μg/kg), Cr (0.5 μg/kg), Cd (0.05 μg/kg), and Ni (0.03 μg/kg). In order to control the accuracy of the sample analysis, the standard reference material of the China National Standard Research Center (GBW07405-yellow/red soil, Hunan) was used and each sample was run as three replicates with reagent blanks monitored as controls. The recoveries for the target PTEs in GBW07405 were good and ranged between 95% and 104%.

## 3. Data Analysis

### 3.1. Geo-Accumulation Index (I_geo_)

The geo-accumulation index (I_geo_) was first proposed by Muller [16] and is currently widely used to assess the level of metal pollution in soils and sediments. It is based on the comparison of the element content in the soil in the study area with the local background value. The formula used was:

(1) $$I_{\mathrm{geo}}=\log_{2}\frac{C_{n}}{1.5C_{r}}$$

where C_n_ is the measured element concentration in the sample. C_r_ is the soil background value of the measured element. This study used the soil background value of Hunan Province [17]. The I_geo_ can be divided into seven categories, from less than 0 to greater than 5 (uncontaminated to extreme pollution) see (Table S1).

### 3.2. Pollution Load Index (PLI)

The pollution load index (PLI) was first proposed by Tomlinson et al. [18] in a screening tool for assessing the pollution level of trace elements. The results of PLI can reflect the enhanced contribution of PTEs to the substance load in the study area. The calculation formula is:

(2) $${\mathrm{CF}}_{n}=\frac{C_{n}}{B_{n}}\;$$

(3) $$\mathrm{PLI}=\sqrt[{n}]{{\mathrm{CF}}_{1}\times {\mathrm{CF}}_{2}\times \cdots \times {\mathrm{CF}}_{n}}$$

where CF_n_ is the pollution factor of element n. C_n_ is the content of element n in the sample. B_n_ is the background value of element n and this study used the soil background value of Hunan Province [17]. CF and PLI classifications are presented in Table S1.

### 3.3. Potential Ecological Risk Index (RI)

The potential ecological risk index (RI) was first proposed by Hakanson [19] in 1980. The RI assesses the ecological risk of PTEs in the soil based on element content and its toxicity [20]. The calculation formula is:

(4) $$\mathrm{RI}=\sum^{\;}E_{r}^{i}=\sum^{\;}T_{r}^{i}\times \left(\frac{C}{C_{n}^{i}}\right)$$

where RI is the sum of the potential ecological risks of various PTEs. $E_{r}^{i}$ is the RI of an individual element. C is the content of PTEs in the sample. $C_{n}^{i}$ is the background value of the element in the soil of Hunan Province. $T_{r}^{i}$ is a toxic response factor of PTEs. The toxic response factors of Mn, Pb, Cu, Zn, Cr, Cd, and Ni are 1, 5, 5, 1, 2, 30, and 5, respectively [19,21,22]. The RI classification criteria for PTEs are found in the Supplementary Information (Table S2).

### 3.4. Health Risk Assessment

The excessive ingestion of PTEs in the human body can cause acute or chronic health problems [23]. The hazard quotient (HQ) is widely used to study the health risks of adults and children in contaminated soil environments [24,25]. In addition, in order to assess the cumulative health risks generated by multiple elements, the HQ values of all PTEs are summed to obtain a comprehensive risk index, called the hazard index (HI) [26]. Carcinogenic risk (CR) is the probability that everyone will get cancer due to exposure to carcinogenic hazards. In the presence of multiple carcinogens, the total carcinogenic risk will increase with each carcinogen and exposure pathway [27,28]. The elements involved in carcinogenic risk in this study are Cr, Cd, and Ni. The calculation formulae for HQ, HI and CR are as follows:

(5) $${\mathrm{HQ}}_{\mathrm{ingestion}}=\frac{C\times \mathrm{IR}\times \mathrm{RBA}\times \mathrm{EF}\times \mathrm{ED}}{\mathrm{BW}\times \mathrm{AT}\times {\mathrm{RfD}}_{o}}\times 10^{-6}$$

(6) $${\mathrm{HQ}}_{\mathrm{dermal}}=\frac{C\times \mathrm{SA}\times {\mathrm{AF}}_{s}\times {\mathrm{ABS}}_{d}\times \mathrm{EF}\times \mathrm{ED}}{\mathrm{BW}\times \mathrm{AT}\times {\mathrm{RfD}}_{o}\times \mathrm{GIABS}}\times 10^{-6}$$

(7) $${\mathrm{HQ}}_{\mathrm{inhalation}}=\frac{C\times \mathrm{EF}\times \mathrm{ED}}{\mathrm{PEF}\times \mathrm{AT}\times \mathrm{RfC}}$$

(8) $$\mathrm{HI}=\sum^{\;}\mathrm{HQ}$$

(9) $${\mathrm{CR}}_{\mathrm{ingestion}}=\frac{C\times \mathrm{IFS}\times \mathrm{RBA}\times {\mathrm{CSF}}_{o}}{\mathrm{AT}}\times 10^{-6}$$

(10) $$\mathrm{IFS}=\frac{\mathrm{EF}\times \mathrm{ED}\left(\mathrm{adult}\right)\times \mathrm{IR}\left(\mathrm{adult}\right)}{\mathrm{BW}\left(\mathrm{adult}\right)}+\frac{\mathrm{EF}\times \mathrm{ED}\left(\mathrm{child}\right)\times \mathrm{IR}\left(\mathrm{child}\right)}{\mathrm{BW}\left(\mathrm{child}\right)}$$

(11) $${\mathrm{CR}}_{\mathrm{dermal}}=\frac{C\times \mathrm{DFS}\times {\mathrm{ABS}}_{d}\times {\mathrm{CSF}}_{o}}{\mathrm{AT}\times \mathrm{GIABS}}\times 10^{-6}$$

(12) $$\mathrm{DFS}=\frac{\mathrm{EF}\times \mathrm{ED}\left(\mathrm{adult}\right)\times \mathrm{SA}\left(\mathrm{adult}\right)\times \mathrm{AF}\left(\mathrm{adult}\right)}{\mathrm{BW}\left(\mathrm{adult}\right)}\;+\frac{\mathrm{EF}\times \mathrm{ED}\left(\mathrm{child}\right)\times \mathrm{SA}\left(\mathrm{child}\right)\times \mathrm{AF}\left(\mathrm{child}\right)}{\mathrm{BW}\left(\mathrm{child}\right)}$$

(13) $${\mathrm{CR}}_{\mathrm{inhalation}}=\frac{C\times \mathrm{EF}\times \mathrm{ED}\times \mathrm{IUR}}{\mathrm{PEF}\times \mathrm{AT}}\times 10^{3}$$

(14) $$\mathrm{TCR}=\sum^{\;}\mathrm{CR}$$

where C is the concentration (mg/kg). RBA (unitless) is the relative bioavailability factor (set to 1 according to USEPA) [29]. RfD_o_ (mg/kg·day) is based on USEPA non-carcinogenic risk oral reference dose [30]. ABS_d_ (unitless) is the dermal absorption rate, the value of which is determined by USEPA [24]. GIABS (unitless) is a gastrointestinal absorption factor, the value of which is determined by USEPA [30]. PEF (m^3^/kg) is a particulate emission factor, the value of which is determined by USEPA [30]. RfC (mg/m^3^) is the inhalation reference concentration, the value of which is determined by USEPA [30]. IFS (mg/kg) is the soil ingestion ratio, the specific value of which is determined by the formula, which is derived from USEPA [29]. DFS (mg/kg) is the soil dermal contact factor, the specific value is determined by the formula, which is derived from USEPA [29]. IUR (μg/m^3^)^−1^) is the inhalation unit risk, the value of which is determined by USEPA [30]. The definition and values of the parameters in the formula are provided in the Supplementary Information (Tables S3 and S4).

When HQ (HI) < 1, it is generally considered health protective. In contrast, when HQ (HI) > 1, and increased health risk may occur, which increases as the HQ (HI) value becomes higher [31]. Similarly, when CR < 10^−6^, there is negligible carcinogenic risk; when 10^−6^ < CR < 10^−4^, there may be carcinogenic risk, but it is acceptable; and when CR > 10^−4^, there is a high carcinogenic risk.

### 3.5. Statistical Analysis

Descriptive statistical analysis of soil properties and PTE content was performed using SPSS22 (IBM, Armonk, NY, USA). The Pearson correlation coefficient matrix and PCA were used to identify the source of PTEs. In addition, they can provide effective information to identify the source of elements in the study area [1,32]. Origin9 was used to convert the data into graphics, making the assessment more intuitive. Kriging interpolation in ArcGIS 10.3 (ESRI, Redlands, CA, USA) can be used to obtain the information relating to the spatial distribution of PTEs.

## 4. Results and Discussion

### 4.1. Pollution and Spatial Distribution of PTEs

#### 4.1.1. Pollution Significance

The average concentrations of Mn, Pb, Cu, Zn, Cr, Cd, and Ni in the soil were 1876.67, 40.76, 45.03, 105.07, 41.24, 0.54, and 30.94 mg/kg, respectively (Table 1), exceeding the background values of these elements in Hunan Province by 4.09, 1.37, 1.65, 1.1, 0.58, 4.29, and 0.97 times, respectively. The coefficient of variation (CV) can reveal the disturbance of the soil properties by inputs from the external environment or natural heterogeneity. In general, the higher the CV value, the more severe the disruption [33]. In this study, the high average concentration, the extreme maximum values and high CV for Mn, Pb, Cu, Zn, and Cd indicate that this area might be impacted by external factors such as anthropogenic interference from mining and traffic-derived emissions.

The concentrations of the PTEs in the soil descended in the order Mn > Zn > Pb > Cu > Cr > Ni > Cd. Comparing this to the soil background values for Hunan Province [17], the percentage of samples with contents of Mn, Pb, Cu, Zn, Cr, Cd, and Ni exceeding their background values were 100%, 81.8%, 89.1%, 43.6%, 0%, 98.2%, and 47.3%, respectively. However, compared with other regions in China, Pb, Cu, Zn, Cr, and Cd in this region were lower than those for example from the thallium mining area of Guizhou [1]. For Pb, Zn, Cr, and Cd, the soil contents in this region were lower than that found in the copper mining area of Henan [34]. Apart from Mn, all other elements in this region were lower than for those in the Dexing Copper mining area of Jiangxi [35]. Compared with other mining areas internationally, such as the Malaysian bauxite mining areas, all elements in this region were higher [36]. Compared with the Nigerian Pb–Zn mining regions, Mn, Cu, Cr, and Ni in this region were at higher concentrations, while Pb, Zn, and Cd were at lower concentrations [37] and compared to an India chromite mining area, all elements except Cr and Ni were at higher concentrations [38] (Table 2). The region therefore shows modest levels of contamination by mining derived emissions and the samples collected in this campaign showing significantly lower levels than those from our study closer to mining and smelting operations in other parts of the region [5].

#### 4.1.2. Spatial Distribution

**Figure 2:**
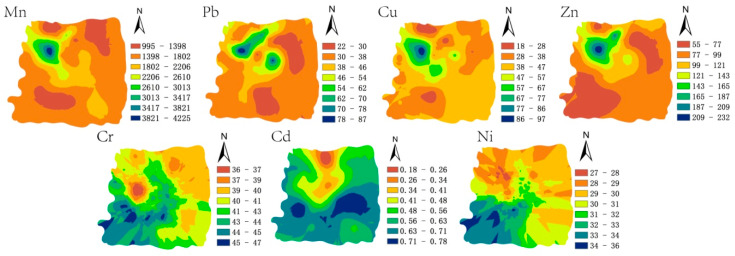
PTE concentration (mg/kg) distribution in the soil of the study area.

In ArcGIS 10.3 (ESRI, Redlands, CA, USA), Kriging interpolation was used to obtain the visual information of the spatial distribution of the seven PTEs (Figure 2). The maximum concentrations of Mn, Pb, Cu, and Zn were all located in the northwest of the study area. Cd was at a high concentration in the south and Cr and Ni were at a high concentration in the southwest (Figure 2). The results of the field investigations revealed that the entire research area was affected by the legacy of direct mining and the impact of smelting and transportation. Frequent mining activities, smelting and transportation may cause changes in the concentration of Mn and other PTEs. In addition, agricultural activities and municipal wastewater production may also interfere with the distribution of Cd and other PTEs in this study area. A large number of herbicides and pesticides used by residents in agricultural activities will also increase the Cd concentration in the soil, and agricultural activities will change soil migration properties. The variation of Cr and Ni was low throughout the study area, and the concentration of Cr was less than the background value, with Ni fluctuating around the background value, indicating that these two elements were likely to be subject to only minor inputs from anthropogenic activity.

### 4.2. Environmental Risk Assessment

#### 4.2.1. Geo-Accumulation Index (I_geo_)

**Figure 3:**
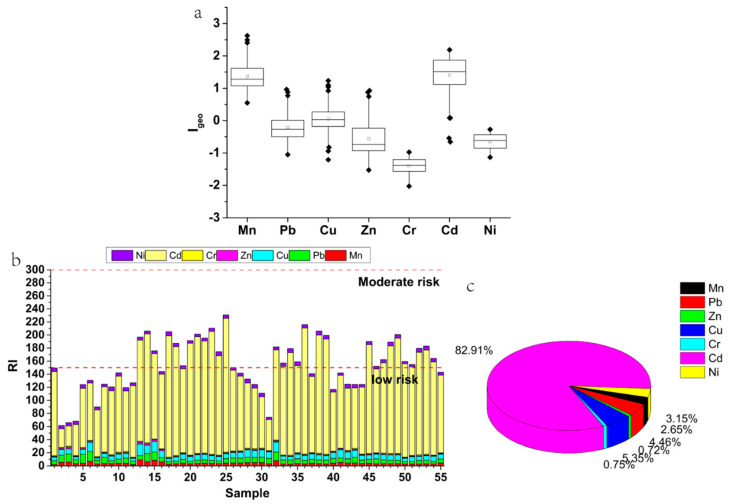
Contribution of the I_geo_ (**a**), potential ecological risk index (RI) of PTEs (**b**) and the $E_{r}^{i}$ of various elements to RI (**c**) in the soil of the study area.

The calculation results showed that the ranges of I_geo_ were from 0.55 to 2.63, −1.05 to 0.97, −1.21 to 1.23, −1.53 to 0.92, −2.02 to −0.97, −0.66 to 2.19, and −1.13 to −0.27 for Mn, Pb, Cu, Zn, Cr, Cd, and Ni, respectively (Figure 3). The average I_geo_ values were −1.40, −0.65, −0.56, and −0.21 for Cr, Ni, Zn, and Pb, respectively (Table 1), and the I_geo_ of all the sampling points were lower than 0 for Cr and Ni, and for Zn and Pb, over 70% of the sampling sites were below 0 (Table S5), so Cr, Ni, Zn, and Pb were uncontaminated in the study area. The average I_geo_ of Cu was 0. 05 and the I_geo_ values for 47.3% of the sampling points were less than 0, and 47.2% of the sampling points were less than 1 (Table S5). Therefore, the Cu data were generally assessed to be at an uncontaminated to moderate contamination level. The average I_geo_ was 1.37 and 1.40 for Mn and Cd, respectively, and more than 70% of the sampling points were classified to be at moderate contamination levels (Table S5). Therefore, the pollution situation in the entire study area reflects a moderate contamination highlighting the potential need for remediation measures to be made.

#### 4.2.2. Pollution Load Index (PLI)

The results of the PLI calculation for PTEs in the soil samples are shown in the Supplementary Information (Figure S1). The range of CF values were 2.19–9.25, 0.72–2.93, 0.65–3.53, 0.52–2.85, 0.37–0.76, 0.95–6.83, and 0.68–1.24 for Mn, Pb, Cu, Zn, Cr, Cd, and Ni, respectively. In addition, the order of average CF for these PTEs was Cd > Mn > Cu > Pb > Zn > Ni > Cr. This indicates that due to human disturbance, the introduction and distribution of these PTEs in the soil are different [39]. According to the PLI classification standard, 92.7% of the sampling points were in the moderate contamination category, and 7.3% of the sampling points were in the considerable contamination category, confirming the result from the calculation of I_geo_.

#### 4.2.3. Potential Ecological Risk Index (RI)

Figure 3 and Table S5 show the results of the $E_{r}^{i}$ and RI calculations for the PTEs in the soil samples. The ecological risks were lower for Mn, Pb, Cu, Zn, Cr, and Ni than for Cd (Figure 3b). Apart for Cd, the maximum and average values were lower than 40 for the $E_{r}^{i}$ for the elements concerned, indicating that the sampling locations are at low ecological risk across the study area. The average $E_{r}^{i}$ of Cd was 127.79, and the maximum value was more than 200, indicating that there is a potentially significant ecological risk. This may be related to the low background value of Cd in Hunan Province and the high toxic response factor of Cd (30). The range and average of RI were 61.43–230.67 and 154.13, respectively, where, 45.5% were at low risk and 54.5% were at medium risk. Compared with other studies, the average values of PI in the Henan copper mining area and Malaysia bauxite mining areas were 244.4 and 44.93, respectively [34,36]. Therefore, the region as a whole is at a moderate ecological risk. Figure 3 also revealed the contribution of various elements to RI. With Cd dominating through a 82.91% contribution to the RI, followed by Cu and Pb, which contributed 5.35% and 4.46%, respectively, the need for Cd to be prioritized is highlighted as found in previous studies [3,34].

### 4.3. Source Analysis

Pearson correlation coefficient matrix, PCA and rotated component matrix have been widely used to determine the source of PTEs in the soil [1,40,41]. The results of the Pearson correlation coefficient matrix reflected the complex relationship between PTEs in the soil samples (Table 3). The correlations between Mn, Pb, Cu, and Zn were strong (r^2^ > 0.5), and Cr and Ni were also strongly correlated (r^2^ = 0.761). However, the correlation between Cd and other elements was low. Strong correlation indicates that these elements may have common sources, similar migration behavior and pathways in the study area [1,42,43].

Kaiser–Meyer–Olkin (KMO) and Bartlett’s spherical tests were performed on the data before the PCA analysis of the PTE content in the soil. The KMO value of the PTEs in the soil was 0.673, and the Bartlett’s spherical test value was 275.48 (df = 21, Sig = 0.000 < 0.001), indicating that the data were suitable for PCA in this study [44]. Three principal components (PCs) (eigenvalues > 1) were obtained from the data for the seven PTEs in the soil samples, explaining almost 88% of the data variability. According to the rotation component matrix and loading diagram (Table 4 and Figure S2), the first principal component (PC1) accounted for ~49% of the total variance, of which high load elements were Mn, Pb, Cu, and Zn. According to field investigations, high concentrations of Mn, Cu, and Zn may be directly derived from manganese ore activities (such as mining, smelting, and tailing deposits) [45]. In addition, Cu may also originate from the wear of tires and brake linings of vehicles. The main source of Pb is likely to be historical emissions from the use of leaded gasoline by traffic [46]. Although China banned the use of leaded gasoline since 2000, recent reports still show elevated Pb in roadside soil [47]. Although the mining areas in the region have ceased production, the effects of mining, smelting, and tailings on soil for many years still exist. In addition, the strong correlations between Mn, Pb, Cu, and Zn and the similar spatial distribution of these elements also indicate that these elements had a common source (Figure 2 and Table 3). Therefore, the first principal component (PC1) can be considered to be derived from mining activities and related transportation emissions.

The second principal component (PC2) accounted for 24.8% of the total variance, where Cr and Ni had high loadings. The sources of Cr and Ni are commonly natural weathering, industrial (electroplate or tannery), and tailings deposits from manganese mining area [48,49]. According to field investigations, there was no electroplating and tannery industry in the study area, although the concentration of Ni at some sampling points was higher than the soil background value in Hunan Province. In this study, overall, the Ni content fluctuated around the soil background value of Hunan Province and the average value was lower than the background value of Hunan Province. The Cr content was lower than the background value for Hunan Province across the whole study area. In addition, the CV were 16.5% and 16.8% for Cr and Ni, respectively, both of which were lower than 30%, indicating that these two elements were less affected by human interference. Combined with the results from the Pearson correlation coefficient matrix, where Cr and Ni have a strong correlation, this indicates that they are likely to have a common source. Therefore, PC2 can be considered to be dominated by natural inputs.

The third principal component (PC3) accounted for 14.5% of the total variance, for which Cd had a high loading. The Cd may have derived from municipal sewage discharge, agricultural production, and atmospheric deposition [50]. The presence of Cd, as an additive, is widely used in pesticides and is well known locally as a natural contaminant in phosphate fertilizers [51,52]. In this study, samples with higher Cd concentrations were from agricultural areas and towns in the south of the study area. Combined with the Pearson correlation coefficient matrix, the correlation between Cd and several other elements was very low, indicating that Cd is different from other elements. Therefore, PC3 can be considered to be derived from agricultural production and municipal sewage discharge.

### 4.4. Health Risk Assessment

#### 4.4.1. Non-Carcinogenic Risk

**Figure 4:**
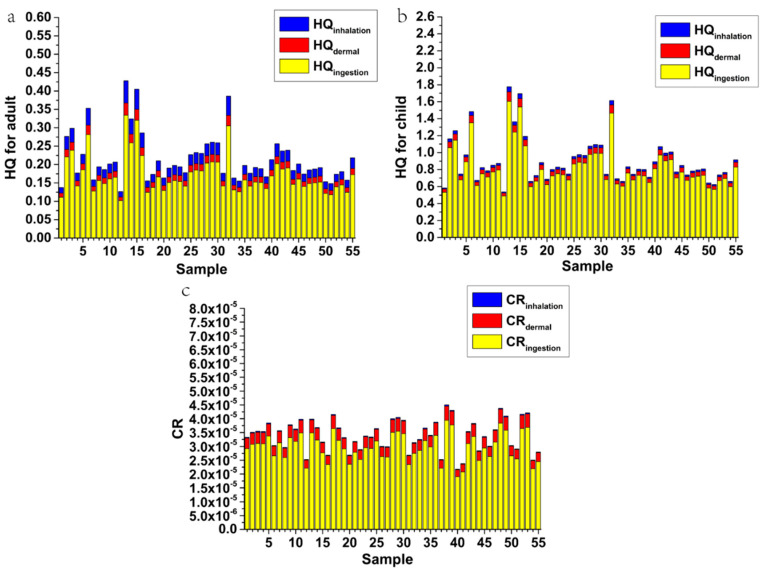
Non-carcinogenic ((**a**): adult and (**b**): child) and carcinogenic risks (CRs) (**c**) of PTEs in soil.

The non-carcinogenic risk analysis for soil sampling sites in the study area for adults and children under different exposure routes is shown in Figure 4 (and Table S6). For adults and children, the relationship between the average non-carcinogenic risk of PTEs in soil was Mn > Pb > Cr > Ni > Cu > Cd > Zn (Table S6). The HQ for all elements was less than one, indicating that these elements do not pose a health risk (Table S6). Similarly, the average values of total HI were 0.215 and 0.905 for adults and children, respectively, which were both lower than one. It shows that there is low toxicity risk across the study area. However, for children, the HI value at some locations was higher than unity, (23.6% of the total sampling points). This shows that whilst overall the region does not present a significant non-carcinogenic risk, this is not uniform and some locations may show significant non-carcinogenic risks for children. These sampling points are mostly located near factories in the north and in urban areas. In addition, Mn has the highest contribution to the total HI, accounting for 76.3% and 73.9% for adult and children, respectively. Figure 4 shows the significant difference between the health risk from PTEs in the soil for adults compared to children, agreeing with previous studies [1,9,53,54]. In addition, the contribution of the three exposure routes to total HI was HQ_ing_ > HQ_inh_ > HQ_der_ (adult), HQ_ing_ > HQ_der_ > HQ_inh_ (child), where, HQ_ing_ accounted for 79.8% (adults) and 90.9% (children) of total HI, respectively.

#### 4.4.2. Carcinogenic Risk

The carcinogenic risk analysis for adults and children under different exposure routes is shown in Figure 4 (and Table S6). The average of the total carcinogenic risk was 3.40 × 10^−5^ for Cr, Cd, and Ni (Table S7). This indicates that the study area may have an increased carcinogenic risk, but it is acceptable. The relationship between the carcinogenic risk of the three exposure routes was CR_ing_ > CR_der_ > CR_inh_ (Figure 4). Therefore, the ingestion route is the main source of carcinogenic risk, accounting for 87.7% of the total carcinogenic risk, dermal contact accounts for 11.5% of the total carcinogenic risk and the contribution of inhalation was insignificant.

### 4.5. Practical Implications of This Study

In this study, the soil from the abandoned manganese ore area in Xiangtan evaluated by I_geo_, PLI, RI, HQ, HI and CR and statisical assessment using PCA and Pearson correlation coefficient. The results highlighted the significance of a number of human inputs which can be used, combined with spatial analysis, to drive the further management of the abandoned region as it is redeveloped., and also be used to analyze the source of soil pollution. According to these results, we can understand the soil pollution in the study area. In the future, the soil will be treated according to its condition, so that it can be re-used better.

## 5. Conclusions

From the assessment of soil contamination in the abandoned mining area:

1. The relationship between the concentration of seven PTEs in the soil system was Mn > Zn > Pb > Cu > Cr > Ni > Cd, where, the concentrations of Mn and Cd, respectively, exceeded their background values by four times. The high average concentration, extreme maximum, and high CV for Mn, Pb, Cu, Zn, and Cd indicate that they have been affected by external sources such as anthropogenic interference (mining and transportation emissions).

The spatial distribution of PTEs showed the north of the region with the most significant concentrations. Calculations of I_geo_, PLI, and RI indicate that across the entire study area, a moderate level of pollution but with some spatial variation including significant peaks.

2. The results of the statistical analysis (Pearson correlation coefficient and PCA) show that PTEs in the survey area are influenced by three discrete sources: mining activity and associated material movement (Mn, Pb, Cu, and Zn), natural geological background (Cr and Ni), and agriculture/municipal waste water (Cd).

3. From the health risk assessment, the non-carcinogenic risk was negligible (HI < 1) across the entire study area, but for children, some locations showed an enhanced risk HI > 1. The HI values for children were significantly higher than for the adults and for the three exposure routes of ingestion, dermal contact, and inhalation, ingestion was the most important. The evaluation of carcinogenic risk showed that there is an acceptable carcinogenic risk and that ingestion was also the main exposure route for carcinogenic risk.

## Figures and Tables

**Table 1:** Potentially toxic elements (PTEs) concentrations and descriptive statistics in the soil (mg/kg).

Element	Minimum	Maximum	Mean ± SD	CV (%)	Background Values	I_geo_
Mn	1005.70	4247.49	1877.67 ± 685.01	36.5	459	1.37
Pb	21.48	87.10	40.76 ± 15.52	38.1	29.7	−0.21
Cu	17.73	96.26	45.03 ± 16.17	35.9	27.3	0.05
Zn	49.08	268.75	105.07 ± 50.73	48.3	94.4	−0.56
Cr	26.34	54.50	41.24 ± 6.81	16.5	71.4	−1.40
Cd	0.12	0.86	0.54 ± 0.17	31.5	0.126	1.40
Ni	21.79	39.57	30.94 ± 5.20	16.8	31.9	−0.65

SD: standard deviation; Geo-accumulation index (I_geo_) is the mean value, CV (%) coefficient of variation.

**Table 2:** Concentrations of soil PTEs (mg/kg) in abandoned manganese mines and other areas in Xiangtan.

	Mn	Pb	Cu	Zn	Cr	Cd	Ni	References
Xiangtan abandoned manganese mine area	1877.65	40.76	45.03	105.07	41.24	0.54	30.94	This study
Guizhou thallium mining area	-	44.39	79.63	118.23	52.11	1.14	-	Jiang et al. [1]
Henan coal mining area	-	70.10	26.97	109.63	50.97	0.61	-	Li et al. [34]
Dexing mining area in Jiangxi	-	98.6	65	134	77.8	1.3	35.9	Lin et al. [35]
Malaysia bauxite mining areas	192.42	13.70	10.73	44.01	34.25	0.13	-	Kusin et al. [36]
Nigeria Pb–Zn mining	788.14	132.4	18.19	134	40.71	0.6	22.23	Obiora et al. [37]
India chromite mining area	-	11.28	24.00	98.54	499.94	-	168.49	Krishna et al. [38]

**Table 3:** Pearson correlation coefficient matrix between PTEs in the soil.

	Mn	Pb	Cu	Zn	Cr	Cd	Ni
Mn	1						
Pb	0.642 **	1					
Cu	0.912 **	0.550 **	1				
Zn	0.874 **	0.694 **	0.731 **	1			
Cr	−0.044	0.018	−0.057	0.003	1		
Cd	−0.183	−0.451 **	−0.099	−0.344 *	0.230	1	
Ni	−0.227	−0.131	−0.241	−0.175	0.761 **	0.183	1

** indicates that the correlation reached a significant level of 0.01 (two-tailed), * indicates that the correlation reached a significant level of 0.05 (two-tailed).

**Table 4:** Total variance and principal component analysis (PCA) of PTEs concentrations in the soil. Strong contribution of components to variability in individual PCs highlighted in **bold**.

Component	Initial Eigenvalues			Element	Rotated Component		
	Total	% of Variance	Cumulative %		PC1	PC2	PC3
1	3.341	49.008	49.008	Mn	**0.974**	−0.075	0.046
2	1.738	24.830	73.838	Pb	**0.739**	0.057	−0.466
3	1.018	14.538	88.377	Cu	**0.922**	−0.110	0.166
4	0.344	4.917	93.294	Zn	**0.909**	−0.001	−0.192
5	0.220	3.145	96.438	Cr	0.034	**0.941**	0.111
6	0.201	2.878	99.317	Cd	−0.192	0.156	**0.930**
7	0.048	0.683	100.000	Ni	−0.187	**0.922**	0.019

## Supplementary Materials

The following are available online at https://www.mdpi.com/1660-4601/17/18/6554/s1, Figure S1: The contamination factors and pollution load index of PTEs in soil, Figure S2: Load diagram in rotating space, Table S1: Levels of I_geo_, CF and PLI; Table S2: Potential ecological risk index classification standard; Table S3: Exposure factors used in estimation for non-carcinogenic risk and carcinogenic risk; Table S4: Some parameter values of various PTEs; Table S5: The distribution of I_geo_ and $E_{r}^{i}$ of PTEs at each level, Table S6: Non-carcinogenic risk hazard quotient (HQ) and risk index (HI), Table S7: Carcinogenic risk under exposure pathways.
