# Supplementary material for: Soil from an Abandoned Manganese Mining Area (Hunan, China): Significance of Health Risk from Potentially Toxic Element Pollution and Its Spatial Context
Source: Int J Environ Res Public Health. 2020 Sep 9;17(18):6554. doi: 10.3390/ijerph17186554 (PMC7558159; doi:10.3390/ijerph17186554)
Supplement: Supplementary file 1 [file ijerph-17-06554-s001.pdf]

Soil from an Abandoned Manganese Mining Area (Hunan, China): Significance of Health Risk from Potentially Toxic Element Pollution and its Spatial Context

Xin Luo, Bozhi Ren, Andrew S. Hursthouse, Jonathan R.M. Thacker and Zhenghua Wang

Figure S1 The contamination factors and pollution load index of PTEs in soil
Figure S2 Load diagram in rotating space
Table S1 levels of I_{geo}, CF and PLI
Table S2 Potential ecological risk index classification standard
Table S3 Exposure factors used in estimation for non-carcinogenic risk and carcinogenic risk.
Table S4 Some parameter values of various PTEs
Table S5 The distribution of I_{geo} and E_r^i of PTEs at each level
Table S6 Non-carcinogenic risk hazard quotient (HQ) and risk index (HI)
Table S7 Carcinogenic risk under exposure pathways

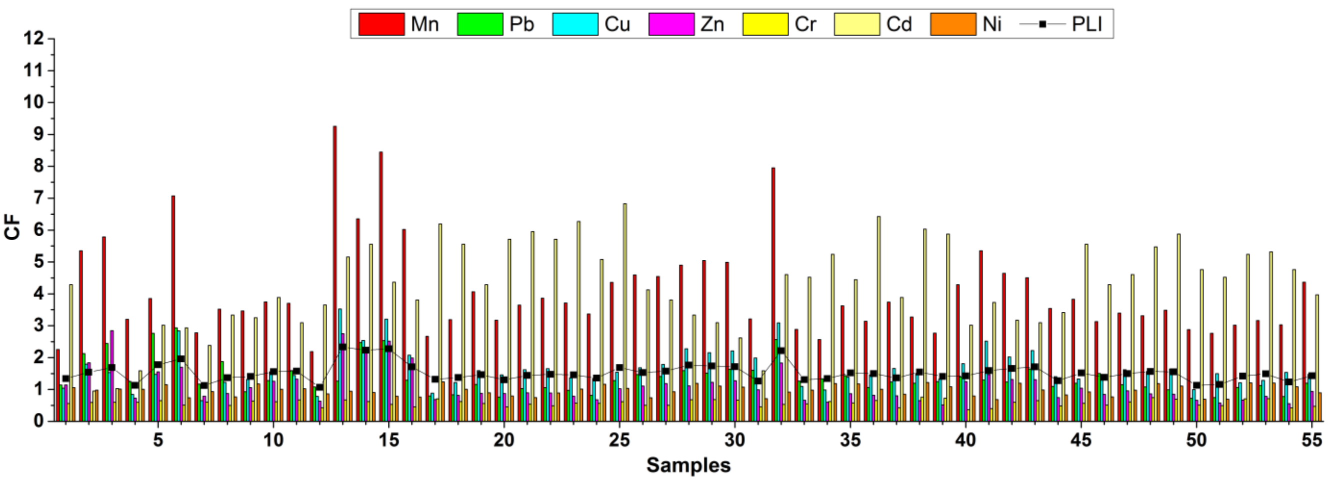

Figure S1 The contamination factors and pollution load index of PTEs in soil

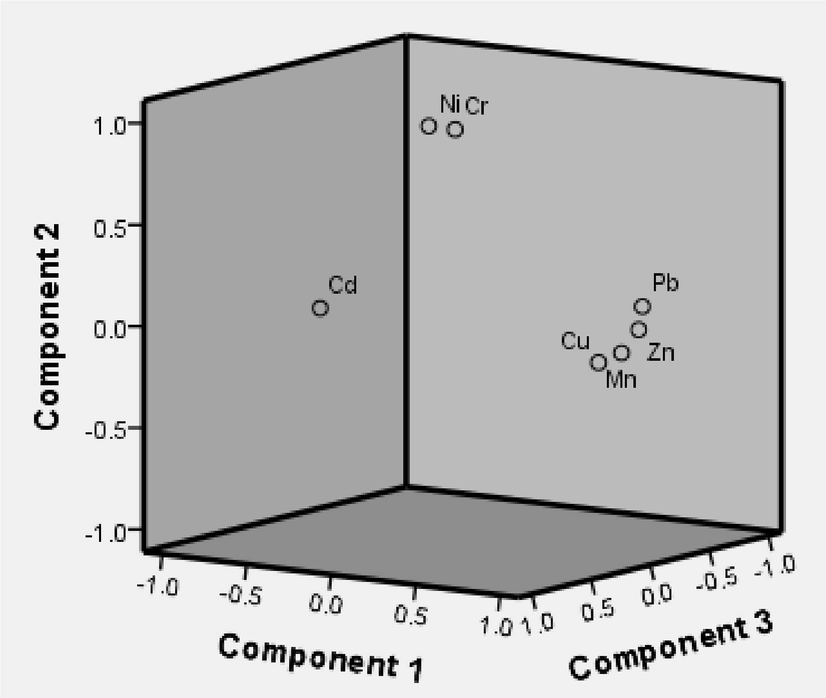

Figure S2 Load diagram in rotating space

Table S1 levels of I_{geo}, CF and PLI

I_{geo}	Pollution level	CF	Pollution level	PLI	Pollution level
I_{geo}≤0	Uncontamination	CF≤1	Low contamination	PLI<1	Low contamination
0<I_{geo}≤1	Uncontamination to moderate contamination	1<CF≤3	Moderate contamination	1≤PLI<2	Moderate contamination
1<I_{geo}≤2	Moderate contamination	3<CF≤6	Considerable contamination	2≤PLI<3	Considerable contamination
2<I_{geo}≤3	Moderate to heavy comtamination	CF>6	Very high contamination	PLI≥3	Very high contamination
3<I_{geo}≤4	Heavy comtamination				
4<I_{geo}≤5	Heavy to extreme contamination				
I_{geo}>5	Extreme contamination				

Table S2 Potential ecological risk index classification standard

E_r^i	Pollution level	RI	Pollution level
E_r^i<40	Low ecological risk	RI<150	Low ecological risk
40 ≤ E_r^i<80	Moderate ecological risk	150≤RI<300	Moderate ecological risk
80 ≤ E_r^i<160	Considerable ecological risk	150≤RI<300	Considerable ecological risk

$160 \leq E_r^i < 320$	High ecological risk	150≤RI<300	High ecological risk
$E_r^i \geq 320$	Very high ecological risk	RI≥600	

Table S3 Exposure factors used in estimation for non-carcinogenic risk and carcinogenic risk.

Variables	Value
IR (mg/day): Soil ingestion rate	100 (adult); 200 (children)
EF (days/year): Exposure frequency	350 days
ED (years): Exposure duration	25 (adult); 8(children)
BW (kg): Average body weight	60 (adult); 25 (children)
AT (days): Averaging time	365×ED adult/children(non-carcinogenic); 365×70 (carcinogenic risk)
SA (cm^2): Skin surface that are available for exposure	5700 (adult); 2800 (children)
AF_s (mg/cm^2): Soil to skin adherence factor	0.07 (adult); 0.2(children)

Adapted from USEPA (1997; 2002; 2009; 2013).

Table S4 Some parameter values of various PTEs

Metal	Relative bioavailability factor(RBA) (unitless)	Dermal absorption fraction (ABS_d) (unitless)	Oral reference dose (RfD_o) (mg/kg·day)	Oral slope factor (CSF_o) $(mg/kg·day)^{-1}$	Gastrointestinal Absorption (GIABS) (unitless)	Inhalation reference concentration (RfC) (mg/m^3)	Particulate emission factor (PEF) (m^3/kg)	Inhalation unit risk (IUR) $(\mu g/m^3)^{-1}$
Mn	1	0.001	2.4×10^{-2}	-	0.04	5.0×10^{-5}	1.36×10^{9}	-
Pb	1	0.001	3.5×10^{-3}	-	1	-	1.36×10^{9}	-
Cu	1	0.001	4.0×10^{-2}	-	1	-	1.36×10^{9}	-
Zn	1	0.001	3.0×10^{-1}	-	1	-	1.36×10^{9}	-
Cr	1	0.001	3.0×10^{-3}	0.5	0.025	1.0×10^{-4}	1.36×10^{9}	8.4×10^{-2}
Cd	1	0.001	1.0×10^{-3}	-	0.025	1.0×10^{-5}	1.36×10^{9}	1.8×10^{-3}
Ni	1	0.001	2.0×10^{-2}	-	0.04	9.0×10^{-5}	1.36×10^{9}	2.6×10^{-4}
References	USEPA[36]	USEPA[31]	USEPA[37]	USEPA[37]	USEPA[37]	USEPA[37]	USEPA[37]	USEPA[37]

Table S5 The distribution of I_{geo} and E_r^i of PTEs at each level

	I_{geo}							E_r^i				
	Class1	Class2	Class3	Class4	Class5	Class6	Class7	Class1	Class2	Class3	Class4	Class5
Mn		16.4%	72.7%	10.9%				100%				
Pb	41.8%	43.7%	14.5%					100%				
Cu	20.0%	56.4%	23.6%					100%				
Zn	80.0%	10.9%	9.1%					100%				
Cr	100%							100%				
Cd	3.6%	9.1%	78.2%	9.1%				3.6%	7.3%	63.6%	25.5%	
Ni	100%							100%				

Table S6 Non-carcinogenic risk hazard quotient (HQ) and risk index (HI)

	Adult				child			
element	HQ_{ing}	HQ_{der}	HQ_{inh}	HI	HQ_{ing}	HQ_{der}	HQ_{inh}	HI
Mn	1.25×10^{-1}	1.25×10^{-2}	2.65×10^{-2}	1.64×10^{-1}	6.00×10^{-1}	4.20×10^{-2}	2.65×10^{-2}	6.69×10^{-1}
Pb	1.87×10^{-2}	7.44×10^{-5}		1.87×10^{-2}	8.96×10^{-2}	2.51×10^{-4}		8.98×10^{-2}
Cu	1.80×10^{-3}	7.18×10^{-6}		1.81×10^{-3}	8.64×10^{-3}	2.42×10^{-5}		8.66×10^{-3}
Zn	5.60×10^{-4}	2.23×10^{-6}		5.62×10^{-4}	2.69×10^{-3}	7.52×10^{-6}		2.69×10^{-3}
Cr	2.20×10^{-2}	3.51×10^{-3}	2.91×10^{-4}	2.58×10^{-2}	1.05×10^{-1}	1.18×10^{-2}	2.91×10^{-4}	1.18×10^{-1}
Cd	8.58×10^{-4}	1.37×10^{-4}	3.78×10^{-5}	1.03×10^{-3}	4.12×10^{-3}	4.61×10^{-4}	3.78×10^{-5}	4.62×10^{-3}
Ni	2.47×10^{-3}	2.47×10^{-4}	2.42×10^{-4}	2.96×10^{-3}	1.19×10^{-2}	8.31×10^{-4}	2.42×10^{-4}	1.29×10^{-2}
Total	1.71×10^{-1}	1.64×10^{-2}	2.70×10^{-2}	2.15×10^{-1}	8.22×10^{-1}	5.54×10^{-2}	2.70×10^{-2}	9.05×10^{-1}

Table S7 Carcinogenic risk under exposure pathways

Element	Cr	Cr	Cr	Cd	Ni	
Pathway	CR_{ing}	CR_{der}	CR_{inh}	CR_{inh}	CR_{inh}	TCR
CR	2.98×10^{-5}	3.90×10^{-6}	2.79×10^{-7}	7.78×10^{-11}	6.48×10^{-10}	3.40×10^{-5}
